# Safety of cannabidiol versus placebo in healthy population: a systematic review and meta-analysis

**DOI:** 10.1097/MS9.0000000000004549

**Published:** 2026-02-12

**Authors:** FNU Sawaira, Muhammad Asim Shah, Ahmed W. Hageen, Maham Rashid, Rahat Ullah, Mohammad Eisa Ali, Aizaz Ali, Muhammad Younas, Hassaan Qazi, Muhammad Owais, Muhammad Yasir, Husna Irfan Thalib, Mazhar Ali Shah, Kamil Ahmad Kamil

**Affiliations:** a Khyber Girls Medical College, Peshawar, Khyber Pakhtunkhwa, Pakistan; b Khyber Medical College, Peshawar, Khyber Pakhtunkhwa, Pakistan; c Faculty of Medicine, Tanta University, Tanta, Egypt; d Dow Medical College, Dow University of Health Sciences, Karachi, Sindh, Pakistan; e Gomal Medical College, Dera Ismail Khan, Khyber Pakhtunkhwa, Pakistan; f Department of Molecular Medicine, University of Pavia, Pavia, Italy; g Khyber Teaching Hospital, Affiliated with Khyber Medical College, Peshawar, Khyber Pakhtunkhwa, Pakistan; h Bannu Medical College, Bannu, Khyber Pakhtunkhwa, Pakistan; i Liaquat University of Medical and Health Sciences (LUMHS), Jamshoro, Sindh, Pakistan; j Bacha Khan Medical College, Mardan, Khyber Pakhtunkhwa, Pakistan; k Batterjee Medical College, Jeddah, Makkah Province, Saudi Arabia; l Internal Medicine Department, Mirwais Regional Hospital, Kandahar, Afghanistan

**Keywords:** adverse effects, cannabidiol, CBD, diarrhea, healthy adults, randomized controlled trials, safety profile

## Abstract

Cannabidiol (CBD) has gained significant attention for its potential therapeutic benefits, but its safety profile in healthy populations remains underexplored. This systematic review and meta-analysis aim to evaluate the safety of CBD compared to placebo in healthy adults. A comprehensive search of PubMed, Embase, Cochrane CENTRAL, and ClinicalTrials.gov identified four randomized controlled trials (RCTs) involving 269 healthy adults. The primary outcome was headache, with secondary outcomes including fatigue, abdominal pain, diarrhea, upper respiratory tract infection (URTI), and dizziness. The results revealed that CBD use was associated with a significantly higher risk of diarrhea (RR = 5.85; 95% CI = 1.14–30.02; *P* = 0.03). While there was a trend toward increased abdominal pain and headache, these results were not statistically significant. Fatigue, dizziness, and URTI showed no significant differences between the CBD and placebo groups. These findings suggest that while CBD appears safe for short-term use in healthy adults, gastrointestinal side effects, particularly diarrhea, should be monitored. Further large-scale studies with longer follow-up periods are warranted to confirm these results and explore long-term safety.

## Introduction

Cannabidiol (CBD) is a nonpsychoactive compound derived from the Cannabis plant. It has garnered significant attention for its therapeutic potential across a range of conditions, including anxiety, epilepsy, and chronic pain^[1]^. CBD acts on various receptors like the Type 1 cannabinoid receptor, Type 2 cannabinoid receptor, GPR55, transient receptor potential vanilloid, and peroxisome proliferator-activated receptor gamma. By modifying the activities of these receptors, the use of CBD has increased globally in recent years, particularly for multiple therapeutic effects^[2,3]^.

**HIGHLIGHTS**

- First meta-analysis on CBD safety in healthy adults.
- Four RCTs with 269 participants.
- CBD significantly increases the risk of diarrhea versus placebo.
- Other adverse effects show no significant differences.
- CBD is generally safe short term but needs GI monitoring.

The therapeutic effects include neuroprotective, antiepileptic, anxiety, antipsychotic, anti-inflammatory, sleep disturbances, stress, analgesic, and anticancer properties^[4,5]^. CBD has gained increasing attention for its potential therapeutic benefits and favorable safety profile compared to Δ9-tetrahydrocannabinol^[6,7]^.

As the popularity of CBD products increases, especially in the form of supplements and over-the-counter remedies, understanding its safety profile in healthy populations is essential. This is important for both consumers and healthcare providers. The current body of research includes studies on CBD’s impact on various clinical conditions. Some studies indicate its ability to reduce seizures in pediatric epilepsy patients and manage anxiety symptoms.^[1,8]^ However, most of these studies focus on specific patient populations, such as those with epilepsy or chronic pain, rather than healthy adults^[9]^. The existing studies have focused on populations with pre-existing conditions such as epilepsy^[10]^, often using high-dose CBD and additional medication that may contribute to hepatotoxicity^[11]^.

The evidence from clinical trials has shown the potential increase in hepatotoxicity associated with CBD use^[12–14]^. Notably, studies investigating high-dose CBD (≥20 mg/kg/day) for treatment-resistant epilepsy in pediatric and adult populations have reported elevated liver enzymes (LEs)^[15,16]^. Recent trials show similar hepatic changes in healthy adult individuals receiving moderate doses of CBD (e.g., 300 mg/day)^[17]^, where some individuals have elevated LEs predictable to the drug-induced liver injury (DILI)^[13]^.

In addition, the previous studies have not assessed safety outcomes in healthy individuals in detail. Therefore, we undertook this systematic review and meta-analysis to evaluate the safety of CBD versus placebo in healthy adult populations. The findings of this review aim to provide evidence for the recommendations of CBD in healthy adults.

This study adheres to the TITAN Guidelines 2025 for transparent and ethical reporting on the use of Artificial Intelligence (AI) in scientific writing and data synthesis. No generative AI tools were used for data analysis, statistical computation, or decision-making processes^[18]^.

## Methodology

### Protocol and reporting standards

This meta-analysis was conducted in accordance with the Cochrane Handbook for Systematic Reviews of Interventions and has been reported in line with the PRISMA (Preferred Reporting Items for Systematic Reviews and Meta-Analyses) 2020 Guidelines to ensure transparency, accuracy, and reproducibility of the methodology and findings^[19]^. The protocol was prospectively registered with PROSPERO (Registration ID: CRD420251113317). This review has also been conducted and reported in accordance with the AMSTAR (Assessing the Methodological Quality of Systematic Reviews) Guidelines, which provide a validated framework for evaluating the methodological rigor, transparency, and reproducibility of systematic reviews.

### Search strategy and data sources

A comprehensive literature search was conducted in PubMed, Embase, Cochrane CENTRAL, and ClinicalTrials.gov from inception to July 2025. The search strategy employed a combination of Medical Subject Headings and key words of the following terms: (Cannabidiol OR CBD OR “medical cannabis”) AND (placebo OR “control group” OR “inactive substance” OR “comparison group” OR “sham treatment” OR “standard care”) AND (Healthy population).

### Eligibility criteria

Studies were included if they met the following criteria:

- population: healthy adults (ages 18–55),
- intervention: CBD in various doses (e.g., 1500–6000 mg, single or multiple doses),
- comparator: placebo,
- outcomes: the following outcomes were reported: diarrhea, fatigue, upper respiratory tract infection (URTI), abdominal pain, headache, and dizziness, and
- study design: randomized controlled trials (RCTs) in English and available in full-text format.

### Outcomes

The primary outcome of our study was headache, while the secondary outcomes included diarrhea, fatigue, URTI, abdominal pain, and dizziness.

Where a change in scores was required, SDs for mean changes were computed using standard formulas. These conversions were performed using Meta-Analysis Accelerator, an online web-based tool^[20]^.

### Study selection

**Figure 1.**
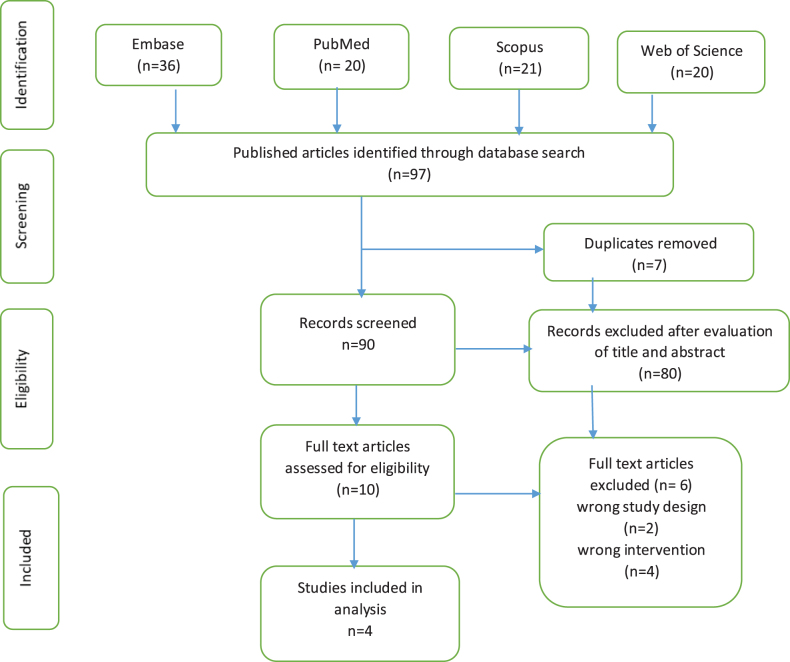
PRISMA flow diagram.

Two independent reviewers (MY, MAS) screened all retrieved titles and abstracts against predefined eligibility criteria. Full texts of potentially eligible studies were reviewed for inclusion. Disagreements were resolved through discussion with a third reviewer (FS). As a result of screening, we finally got four RCTs^[21–24]^. The study selection process is detailed in the PRISMA flow diagram (Fig. 1).

### Data extraction

Data were extracted independently by three reviewers (FS, AA, HQ) using a pre-designed Excel spreadsheet. Extracted variables included sample size, participant age (mean ± SD), gender distribution, BMI (mean ± SD), and race/ethnicity. Follow-up periods ranged from 7 to 35 days. Extracted outcome data for all adverse events across the included trials are summarized in Supplemental Digital Content Table S2, available at: http://links.lww.com/MS9/B93.

### Quality assessment and risk of bias

Risk of bias was assessed independently by two reviewers using the Cochrane Risk of Bias 2.0 (Rob 2.0)^[25]^ tool, evaluating five domains: bias arising from the randomization process, deviations from intended interventions, missing outcome data, measurement of outcomes, and selection of reported results. Discrepancies were resolved by consensus. All four RCTs were judged to have minimal risk of bias across all five domains.

### Statistical analysis

All statistical analyses were performed using Review Manager (RevMan) version 5.4.1. For dichotomous outcomes, pooled risk ratios (RR) with 95% confidence intervals (CI) were calculated using the Mantel–Haenszel method.

Heterogeneity was assessed using the Chi^2^ test and *I*^2^ statistic. *I*^2^ values of 25, 50, and 75% were interpreted as low, moderate, and high heterogeneity, respectively. Due to the small number of studies per outcome (<10), funnel plots and Egger’s test for publication bias were not performed.

## Result

### Study characteristics

This systematic review and meta-analysis included 4 RCTs with 269 healthy adults (ages 18–55), primarily with a BMI of 23–30 kg/m^2^. Sultan *et al* (2019) involved 13 males, mean age 26.3 years; Perkins *et al* (2020) included 24 participants (18–48 years); Taylor *et al* (2018) had 24 adults (18–45 years); and Floorian *et al* (2025) recruited 201 adults with a median age of 36 years. Baseline demographic and clinical characteristics of the included studies are summarized in Supplemental Digital Content Table S1, available at: http://links.lww.com/MS9/B92.

All participants were healthy with no significant medical conditions or recent cannabis use. Full details of the study and patient characteristics are provided in Supplemental Digital Content Table S1, available at: http://links.lww.com/MS9/B92 and Supplemental Digital Content Table S2, available at: http://links.lww.com/MS9/B93.

### Meta-analysis of outcomes

#### Primary outcome

1. **Headache**

**Figure 2.**
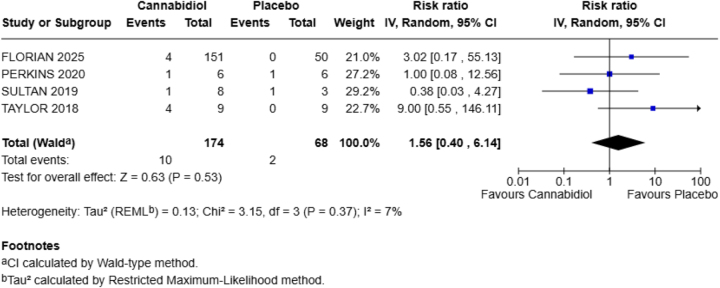
Forest plot for headache (primary outcome).

This outcome was reported by all four included studies in our meta-analysis. Compared to the placebo group, CBD increases the chances of this adverse effect by 56% in the healthy population (RR = 1.56; 95% CI = 0.40–6.14). The *P*-value is nonsignificant (*P* = 0.53) with heterogeneity (*I*^2^ = 7%) (Fig. 2).

#### Secondary outcomes

1. **Fatigue**

Fatigue was the reported outcome by three out of four included RCTs in our meta-analysis. The aggregated findings indicated that RR = 0.62 with a 95% CI of 0.13–2.87, showing that CBD decreases the risk of fatigue by 38% in the exposed group.

**Figure 3.**
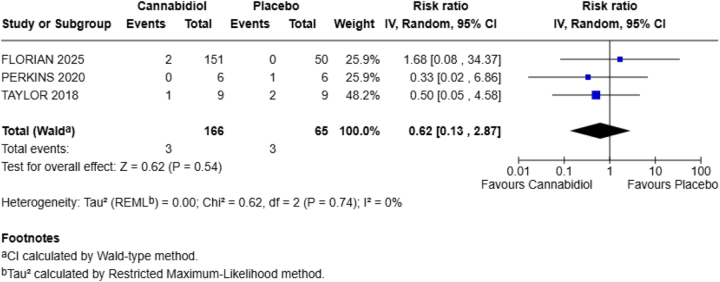
Forest plot for fatigue.

There was no statistically significant difference between the two groups for the occurrence of this adverse effect in the healthy population, as denoted by the *P*-value (*P* = 0.54). No heterogeneity was reported (Fig. 3).

1. **Abdominal pain**

**Figure 4.**
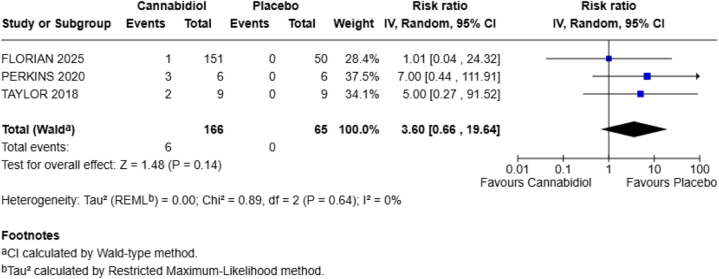
Forest plot for abdominal pain.

Three out of four included RCTs reported this outcome. The combined results were RR = 3.60; 95% CI = 0.66–19.64; *P* = 0.14 with no heterogeneity (*I*^2^ = 0%), showing that CBD increases the chances of abdominal pain in the healthy population by 260% and the *P*-value is statistically nonsignificant (Fig. 4).

1. **Diarrhea**

Three of the included studies in our meta-analysis reported diarrhea as one of their outcomes.

**Figure 5.**
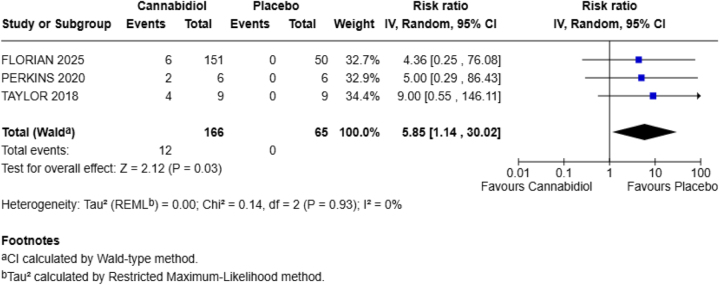
Forest plot for diarrhea.

CBD increases the chances of diarrhea in our population as compared to the placebo group (RR = 5.85; 95% CI = 1.14–30.02). The *P*-value shows statistically significant results (*P* = 0.03) with no heterogeneity (*I*^2^ = 0%) (Fig. 5).

1. **Upper respiratory tract infection**

URTI was reported by three of the four RCTs included in our meta-analysis. Compared to the placebo group, CBD did not show significant results denoted by the *P*-value (*P* = 0.65), with heterogeneity (*I*^2^ = 8%).

**Figure 6.**
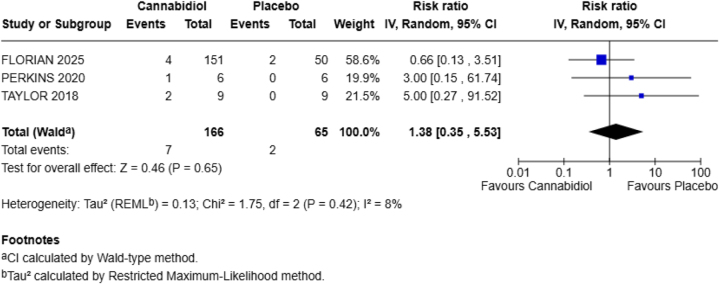
Forest plot for upper respiratory tract infection (URTI).

Other findings show that CBD increases the chances of URTI by 38% (RR = 1.38; 95% CI = 0.35–5.53) (Fig. 6).

1. **Dizziness**

This adverse effect was also reported by three of the four included studies in our meta-analysis.

**Figure 7.**
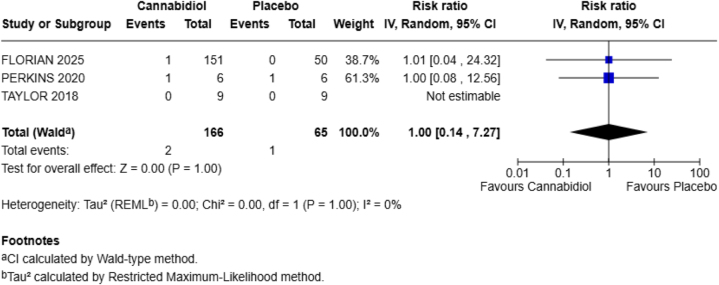
Forest plot for dizziness.

There was no risk of this adverse effect between the two groups (RR = 1.00; 95% CI = 0.14–7.27), showing that CBD neither increased nor decreased the chances of dizziness in our population. The *P*-value is nonsignificant (*P* = 1.00) with no heterogeneity (*I*^2^ = 0%) (Fig. 7).

**Figure 8.**
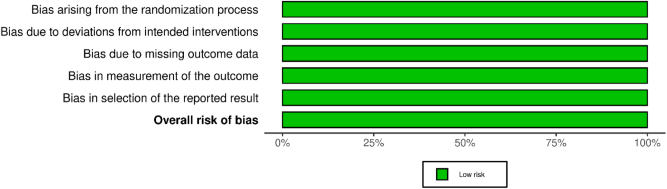
Risk of bias assessment using the Cochrane RoB 2.0 tool.

Figure 8 illustrates the distribution of risk of bias across five domains – randomization process, deviations from intended interventions, missing outcome data, measurement of outcomes, and selection of reported results for the included RCTs.

All domains demonstrated a low risk of bias, indicating high methodological quality across the studies.

**Figure 9.**
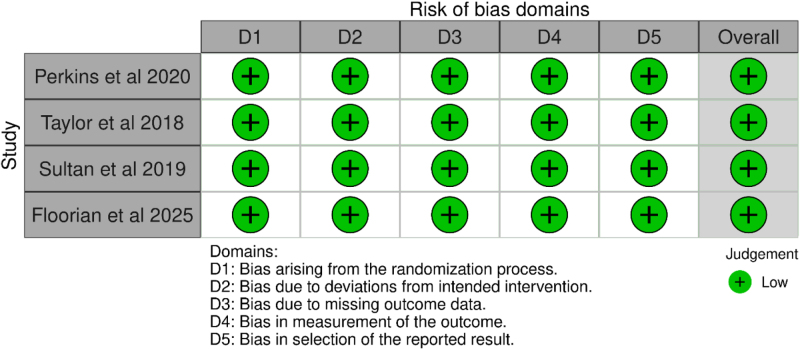
Risk of bias assessment using the Cochrane RoB 2.0 tool.

Figure 9 presents a study-level summary of risk of bias judgments. Each included RCT (Perkins *et al*, 2020; Taylor *et al*, 2018; Sultan *et al*, 2019; and Floorian *et al*, 2025) was independently assessed across all five domains, with all studies rated as having a low risk of bias.

These assessments support the overall robustness and credibility of the meta-analytic findings.

## Discussion

CBD has gained widespread attention in recent years due to its potential therapeutic benefits in a range of medical conditions, such as epilepsy^[26,27]^ and chronic pain^[28,29]^. However, despite its growing popularity and perceived safety, concerns about its side effects, particularly those affecting the liver and gastrointestinal system, are becoming more prominent^[14,30–32]^. Therefore, it is necessary to evaluate its safety profile thoroughly, as CBD becomes increasingly popular for both medical and recreational use. Our systematic review and meta-analysis aimed to assess the adverse effects related to CBD use by analyzing data from 4 RCTs involving 269 healthy individuals.

Earlier investigations have demonstrated that CBD use is significantly associated with increased risks of LE elevation and DILI compared to placebo nine. Notably, DILI was not reported in adults taking CBD doses below 300 mg/day^[11]^. This is crucial in clinical practice, as most individuals using CBD for medical purposes take doses below this threshold^[5,33,34]^. The incidence rates were 7.4% for LE elevation and 2.96% for DILI in people taking CBD 9, classifying them as common adverse events according to CIOMS classification^[35]^. Furthermore, the incidence of CBD-related DILI is similar or exceeds that of known hepatotoxic medications, such as fluoroquinolones, valproic acid, and statins^[36]^. Importantly, no severe DILI cases were reported in prior evidence^[37,38]^.

Our study examined six adverse effects, including headache, fatigue, abdominal pain, diarrhea, URTIs, and dizziness. Among these, only diarrhea showed a statistically significant increase in the CBD group. Specifically, the risk of diarrhea was six times higher in the CBD group compared to placebo (RR = 5.85; 95% CI = 1.14–30.02; *P* = 0.03). There was no statistical heterogeneity (*I*^2^ = 0%), which suggests consistency among the included studies. Other side effects, such as headache (RR = 1.56), fatigue (RR = 0.62), abdominal pain (RR = 3.60), URTI (RR = 1.38), and dizziness (RR = 1.00), were not significantly different between the CBD and placebo groups.

Among all the reported outcomes, diarrhea was the most clearly linked to CBD use. This finding is supported by previous clinical trials, especially those evaluating high doses of CBD in the treatment of epilepsy. For instance, Taylor *et al*^[23]^ reported that the incidence of diarrhea was high with the use of CBD (67% subjects taking CBD vs. 0% subjects taking a placebo) is consistently reported as one of the most common side effects in these settings.

Similarly, Florian *et al*^[39]^ demonstrated that CBD use was associated with several adverse events, including diarrhea (8% of participants taking CBD). Besides, a recent trial found that the most frequently reported adverse events linked to CBD use was diarrhea (8%)^[22]^. Moreover, in patients with epilepsy taking up to 50 mg/kg/day CBD, the most common side effects were somnolence, decreased appetite, diarrhea, pyrexia, and fatigue^[15,16,39,40]^. In tuberous sclerosis complex patients taking up to 50 mg/kg/day CBD, the most common side effects were drowsiness, ataxia, and diarrhea^[41]^. The exact cause of diarrhea is not entirely understood, but explanations include CBD’s interaction with the enteric nervous system, changes in gut motility^[31,42]^, or its effects on bile acid metabolism^[43]^. The statistically significant increase in diarrhea observed in our study suggests that even healthy individuals, without underlying disease, may experience this adverse effect, especially at higher doses.

Abdominal pain also appeared more frequently in the CBD group, with a relative risk of 3.60. Although this did not reach statistical significance (*P* = 0.14). This was consistent with prior evidence that reported a comparable incidence of abdominal pain between CBD and placebo^[33]^. Another study reported that one participant (0.7%) had ALT >18× the upper limit of normal with concurrent symptoms, specifically temporary epigastric discomfort 18. This could suggest that CBD irritates the gastrointestinal tract or alters digestion in ways that lead to discomfort or cramping. Importantly, the studies that reported this outcome showed no heterogeneity (*I*^2^ = 0%), underscoring a consistent finding across the trials. The lack of significance may be due to the small sample sizes, and larger studies could potentially detect a clearer association.

Furthermore, headache was another reported outcome with a relative risk of 1.56, indicating an increase in the CBD group. However, this result was not statistically significant (*P* = 0.53, reflecting uncertainty. In previous literature^[21,22]^, headache has been noted in some CBD trials. Sultan *et al*^[21]^ revealed that post-CBD administration was associated with many side effects, including headache on day 3 (*n* = 1; 3.8%). Perkins *et al*^[22]^ reported that one of the most frequently reported treatment-emergent adverse events was headache (17%). Interestingly, fatigue was reported less often in the CBD group, with a relative risk of 0.62. This finding was aligned with a recent trial^[22,39]^. This suggests that CBD might have a mild protective effect against fatigue. However, this finding was not statistically significant (*P* = 0.54).

In contrast, another study demonstrated that one of the most frequently reported side effects of CBD was fatigue^[32]^. Additionally, populations with neurological disorders, such as epilepsy, have reported fatigue as a common side effect accompanied by CBD use^[15,16,39,41]^. These variations might be justified by differences in patient demographics, CBD dosages, or study protocols.

Dizziness was equally reported in both groups (RR = 1.00), with no statistical difference (*P* = 1.00), which was aligned with prior research^[22,23]^. This suggests that CBD did not influence the risk of dizziness in our healthy population. Furthermore, URTI showed a relative risk of 1.38 in the CBD group. Nevertheless, the result was not statistically significant (*P* = 0.65). Notably, a recent trial demonstrated that one of the most common adverse events in the CBD group was URTI (18%)^[24]^. Another study reported the presence of infections and infestations^[23]^ and URTI^[22]^ among participants receiving CBD. While this outcome is not related to liver function or gastrointestinal symptoms, we included this outcome to address the broader systemic impact of CBD. However, our findings do not provide enough evidence to reinforce any substantial association between CBD and increased risk of infections.

### Strengths and limitations

The strength of our study is the inclusion of data from only RCTs to provide a comprehensive assessment of the efficacy of CBD, which confers high-quality evidence and mitigates the risk of bias. Besides, this study is the most comprehensive meta-analysis on the topic up to now. Additionally, our focus on healthy individuals eliminates confounding factors associated with underlying diseases or concurrent medications. The application of a random-effects model addressed potential discrepancies across studies, validating a more reliable integration of evidence. The consistent reporting of outcomes across studies and the low heterogeneity in most results support the credibility of our results. However, the application of robust statistical methodologies, such as the adherence to PRISMA guidelines, potentially strengthens the reliability of our findings. Besides, the risk of bias assessments (ROB-2) demonstrated minimal risk of bias in all studies. Lastly, the application of relative risks and CIs enabled us to present a clear and quantitative picture of the adverse event profile of CBD use.

Nevertheless, our study has certain limitations that should be acknowledged. First, the number of included studies is small, and several outcomes were reported by only three trials. This limits the statistical power and increases the risk of false-negative findings. Moreover, all studies involved healthy participants, which limit the generalizability of the findings to patients with chronic illnesses, who are more likely to use CBD as a therapeutic option. There were also differences in CBD dosage, duration of exposure, and formulation across the included trials. These differences could have influenced the findings. Furthermore, the subgroup analysis and assessment of dose–response associations were limited by varying cutoffs and a lack of detailed data across studies. Additionally, the short follow-up periods in most studies prevented us from drawing conclusions regarding long-term safety or delayed complications.

### Clinical implications and future directions

From clinical perspectives, the significant increase in diarrhea underscores the need for physicians to monitor gastrointestinal side effects in individuals using CBD, especially at higher doses. The trend toward increased abdominal pain also substantiates this recommendation. Moreover, patients should be counselled about these potential complications, particularly when starting CBD or increasing the dose. Besides, the lack of significant differences in headache, fatigue, dizziness, and URTI may be reassuring, at least in healthy individuals. However, we recommend taking caution when prescribing CBD to individuals with other medical conditions until more evidence becomes available. Upcoming investigations should focus on large, long-term RCTs in diverse demographics to better understand the safety of CBD. Studies should evaluate different doses, routes of administration, and combinations with other drugs. In addition, standardized approaches for reporting side effects should also be adopted to allow better comparisons across evidence. Importantly, more research on how CBD affects LE, gut function, and immune response could provide valuable insights into the biological basis of these side effects. Finally, real-world data from observational studies could complement RCT findings and help detect rare or long-term complications.

## Conclusion

This study shows that CBD use in healthy individuals is potentially associated with an increased risk of diarrhea. There is also a notable trend toward increased abdominal pain, though the evidence is not yet conclusive. Other complications, such as headache, fatigue, dizziness, and URTI, were not significantly different from placebo.

While CBD appears safe in the short term for healthy people, the gastrointestinal side effects should not be overlooked. Lastly, we support careful monitoring, patient education, and further high-quality investigations to ensure the safe use of CBD across broader demographics.

## Data Availability

The datasets used and/or analyzed during the current study are available from the corresponding author on reasonable request but are available in the public domain and referenced.

## Research registration unique identifying number (UIN)

PROSPERO (Registration ID: CRD420251113317).
